# Diagnostic Value of Amsel’s Clinical Criteria for Diagnosis of *Bacterial Vaginosis*

**DOI:** 10.5539/gjhs.v7n3p8

**Published:** 2014-10-28

**Authors:** Farnaz Mohammadzadeh, Mahrokh Dolatian, Masoome Jorjani, Hamid Alavi Majd

**Affiliations:** 1 Department of Midwifery, International Branch, Shahid Beheshti University of Medical Sciences, Tehran, Iran; 2 Department of Reproductive Health and Midwifery, Faculty of Nursing and Midwifery, Shahid Beheshti University of Medical Sciences, Tehran, Iran; 3 Department of Pharmacology, School of Medicine, Shahid Beheshti University of Medical Sciences, Tehran, Iran; 4 Department of Biostatistics, Faculty of Paramedicine, Shahid Beheshti University of Medical Sciences, Tehran, Iran

**Keywords:** *BV*, Amsel criteria, Nugent scoring system, Gram staining

## Abstract

**Introduction:**

*Bacterial vaginosis* (*BV*) is one of the most prevalent infections in women of reproductive age. Amsel’s criteria and Nugent scoring system are among the most commonly used diagnostic methods. Although Nugent scoring system is considered the gold standard for diagnosing *BV*, it is time consuming and costly, and its interpretation needs lab equipment and experts. Hence, most physicians are inclined to use simpler clinical criteria that are yet accurate instead. The present study aimed to determine the diagnostic value of Amsel’s criteria in diagnosing *BV*.

**Material and Methods:**

This present study was conducted to validate diagnostic tests of *BV* in 120 married women in 2013. Amsel’s criteria and Nugent scoring system were used to diagnose *BV*. Nugent scoring system was considered the gold standard and sensitivity, specificity, positive predictive value and negative predictive value of Amsel’s criteria were compared with those of Nugent scoring system.

**Results:**

Kappa coefficient was used to assess the diagnostic value of Nugent scoring system and Amsel’s criteria. Kappa coefficient was found 0.8, which confirms the reliability of both diagnostic methods. McNemar test did not reveal a significant difference between Nugent scoring system and Amsel’s criteria in terms of diagnosing *BV*. As compared to Nugent scoring system, Asmel’s criteria enjoy sensitivity of 0.91, specificity of 0.91, positive predictive value of 0.86, negative predictive value of 0.94, and accuracy of 0.91.

**Conclusion:**

If lab equipment is not available for diagnosing *BV*, Amsel’s criteria can be as good as Nugent scoring system at diagnosing this infection.

## 1. Introduction

*Bacterial vaginosis (BV)* is one of the most prevalent causes of vaginal secretions during reproductive ages. One-third of women with vaginosis have *BV* (Bohbot, Sednaoui, Verriere, & Achhammer, 2012). The prevalence of *BV* differs depending on geographical location, socio-economic status and ethnicity, from 8% to 51% (Haltas, Bayrak, & Yenidunya, 2012). CDC has reported *BV* as 29.2% in American women aged 14–49 years old, and 25% among pregnant women (CDC, 2010). In Iran, it has been reported from 16.2% to 39.9% (Borjian, Shojaei, Shabanian, & Deris, 2002; Khoushkholgh, Masiha, & Asmar, 2007; Amini, Baghchesaraie, & Torabi, 2009; Shobeiri & Nazari, 2006). This infection is caused by a reduction in H_2_O_2_ producing lactobacilli, and an increase in anaerobic organisms such as *gardenerellavaginalis, mycoplasma huminis* and *prevotellaspecies* (Swidsinski et al., 2013). *BV* has several adverse effects such as amniotic fluid infection, chorioaminonitis, premature rupture of membranes, low birth weight, premature brith, increased incidence of pelvic infection after abortion, vaginal cuff cellulitis after hysterectomy, endometritis, cervicitis, urinary tract infection, cervical intraepithelial neoplasia, increased risk of HIV, increased probability of ectopic pregnancy, infertility, and chronic pelvic pains (Marrazzo, 2013). This infection is asymptomatic in 50%-75% of cases and the symptomatic cases present at homogeneous grayish white smelly secretions, fishy smell after intercourse or during mense (Decherney, Nathan, Laufer, & Roman, 2013). Amsel’s criteria and Nugent scoring system are the most common diagnostic methods for *BV* (Rangari Amit, Parmjit, & Sharma, 2013). Nugent scoring system, developed by Nugent et al. in 1991, is based on gram staining and observing the number of lactobacilli and other morphotypes (different shapes of gardenerellavaginalis, prevotella species, and mobiluncus) which are scored between 0 and 10, where scores 7–10 show *BV*. Its high sensitivity has led to its recognition as the gold standard of *BV* (Nugent, Krohn, & Hillier, 1991).

Amsel et al. (1983) introduced a criterion for diagnosing *BV* in 1983. They stated that the presence of 3 of the following four criteria shows disgnosis of *BV*:

-Increased homogeneous thin vaginal discharge;
-pH of the secretion greater than 4.5;
-Amine odor when potassium hydroxide 10% solution is added to a drop of vaginal secretions;
-Presence of clue cells in wet preparations (Amsel et al., 1983).

Taj et al. examined Amsel’s criteria and other microbiological methods to diagnose *BV* and showed that Amsel’s criteria are acceptable for diagnosing *BV* (Taj, Nasir, Kahkashan, & Anjum, 2012). Rangari et al. in their study of 2013 reported Nugent scoring system had a higher sensitivity in diagnosing *BV* while Amsel’s criteria had less sensitivity and higher specificity. They concluded Amsel’s criteria without utilizing staining methods could be misleading (RangariAmit, Parmjit, & Sharma, 2013). Menard et al. investigated PCR, Amsel’s criteria and Nugent scoring system and reported complete agreement between Nugent scoring system and Amsel’s criteria (kappa valu=0.81, 95% confidence interval 0.70–0.81) (Menard et al., 2010). Nowadays, almost all outpatients are treated based on clinical sings. Failure to use lab methods and microscopic studies like preparing wet slide, measuring pH of vaginal discharges often lead to incorrect diagnosis. It is recommended that these methods be used to increase accuracy of diagnosing vaginitis to a reasonable level. Although Nugent scoring system is the gold standard for diagnosing *BV*, it is time consuming and costly, and its interpretation needs lab equipment and specialists. Furthermore, its report might not be ready timely to help clinical diagnosis of *BV*, therefore, most physicians prefer to use simple accurate clinical criteria (Gutman, Peipert, Weitzen, & Blume, 2005). Given the high prevalence of *BV*, complications of failure to timely diagnose and treat it, and given that it is not always possible to use lab assessment for diagnosis, this study was conducted to determine the diagnostic value of Amsel’s criteria for *BV* in women presenting to Resalat Health Center affiliated to Mazandaran University of Medical Sciences in 2013.

## 2. Method and Materials

The present study is an experimental study to validate diagnostic tests conducted on 120 married women presenting with chief complaints of vaginal discharge, burning or itching to Resalat Health Center in Amol, affiliated to Mazandaran University of Medical Sciences, Iran, from July 2013 to January 2014. The inclusion criteria were: age 18–44 years old, not pregnant, no recent use of oral contraception pills, no vaginal bleeding, no intercourse in the past 48 hours, no antibiotic therapy in the past 2 weeks, no vaginal medication in the past 3 days, no participation in other studies in the past 4 weeks, no early menopause and no mental retardation. Given the different sensitivities of Amsel’s criteria in different studies, the sample size was calculated as 93, using the following formula and considering confidence interval of 95% and maximum error of 10%:


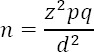


Study population was the married women 18–44 years old who presented to Resalat Health Center with the chief complaints of vaginal discharge, burning or itching. The interested women were briefed on the study and its objectives. A questionnaire on demographic, background and confounding variables was completed to select samples in four sections of demographics, mense and pregnancy history, medical history and health information. After taking history, the participants lay in lithotomy position for physical examination and sampling. Vulva was inspected and a sterile speculum was inserted into the vagina without lubricant. The vagina and cervix were inspected for inflammation, redness, abnormal findings, and discharges were inspected in terms of form, color, consistency and odor. If the patient had signs of pelvic inflammatory diseases or cervicitis, or any other apparent vaginal infections like *trichomonasis* or *candida*, they were excluded. The acidity of the vaginal discharges was measured and recorded using pH paper (Merck, Germany, range 0–14) by placing the paper on the lateral wall of vaginal for one minute (so that it was not in contact with alkaline discharges of cervix). Then, a sterile cotton swab was used to sample discharges from lateral walls and posterior fornix of vagina and immediately transferred to 3 slides. The first slide was examined for trichomona and clue cells by microscope after adding 1–2 drops of normal saline. The second slide was examined using Whiff test by adding a drop of KOH 10% solution and checking for mycelium and hyphae of candida. The clinical criteria for diagnosing *BV* were the presence of three of the Amsel’s criteria (pH≥4.5, positive whiff test, grayish white homogeneous discharge and presence of clue cells). The third slide was air dried, gram stained and sent to lab for microscopic examination (x 1000, using immersion oil) by a pathologist who was blind to the clinical status of patients. The slides were interpreted for *BV* using gram staining and Nugent scoring system. Lab reports were ready within 48 hours. Scoring system ranges from zero to ten, with scores above seven to mark diagnosis of *BV* (Table 1). The data were analyzed using descriptive statistics (mean±SD) and frequency distribution in the SPSS 16 software. The present study considered Nugent scoring system as the gold standard for diagnosis of *BV*, and accordingly the sensitivity, specificity, positive predictive value and negative predictive value of Amsel’s criteria were determined. In all tests, confidence interval of 95% and significance level of 0.05 were considered.

**Table 1:** Nugent scoring system

Mobiluncus Curvedshapes Gram-negative	*Gardnerella, Bacterioid Coccobacillus,* gram-negative with vacuole	Lactobacillus, Gram-positive	score
0	0	>30	0
1-5	<1	5-30	1
>5	1-4	1-4	2
	5-30	<1	3
	> 30	0	4

## 3. Results

The present study recruited 120 married women whose demographic and obstetric characteristics are presented in Table 2. The subjects’ mean age and duration of marriage was 29.97±5.93 (range: 18–44) and 11.25±6.47 years, respectively. Their mean number of pregnancies and abortions was 1.25±1.77 and 0.74±0.25, respectively. The mean frequency of sex among the participants was 1.72±0.88 times per week. The majority of the studied women were housewives (79.2%) and had junior high or high school education (66.7%). Natural birth control method was the most common type of contraception used by 56.2% of the participants.

**Tablet 2:** Frequencydistribution of age, length of marriage, education, occupation and contraception among women attending research in Resalat Health Center of Amol (July 2013 - January 2014)

(%) age			(%) Length of marriage			(%) Education			(%) Occupation			(%) Contraception						

Under 25 years	26-35	Above 36 years	Under 10 years	11-19	Above 20 years	Elementary Education	Secondary education	High School	collegiate	housekeeper	Employed	OCP	Ampul	Condom	WD	TL	IUD	Nothing
25.8	55	19.2	40	45.8	14.2	15	21.7	45	18.3	79.2	20.8	8.6	1	22	56.2	2.9	2.7	6.6

IUD - Intrauterine Device; TL - Tubal Ligation; WD – Withdrawal; OCP - Oral contraceptive pill.

The minimum calculated kappa coefficient between the Nugent scoring system (gold standard) and the Amsel’s criteria (0.8) confirmed the reliability of both diagnostic methods. Moreover, McNemar test did not reveal any significant differences between the two methods in terms of *BV*. The sensitivity, specificity, positive predictive value, negative predictive value, and accuracy of Amsel’s criteria were computed as 91%, 91%, 86%, 94%, and 91%, respectively (Table 3).

**Table 3:** Comparison of Amsel’s criteria and Nugent scoring for the diagnosis of *bacterial vaginosis* among women attending research in Resal at Health Center of Amol (July 2013 - January 2014.)

Nugent scoring					p-value	SN (%)	SP (%)	PPV (%)	NPV (%)	ACCURACY (%)

		Positive	Negative	Total						
Amsel’s criteria	Positive	42	7	49	0.549	0.91	0.91	0.86	0.94	0.91
	Negative	4	67	71						
	Total	46	74	120						

N - Number; SN - Sensitivity; SP - Specificity; PPV - Positive predictive value; NPV - Negative predictive value.

As Table 4 shows, the presence of clue cells on vaginal wet mount had the highest sensitivity among all Amsel criteria. Homogeneous discharge and pH≥4.5 had the second and third highest sensitivity (86.7% and 83.3%, respectively) in the diagnosis of *BV*. Although whiff test had the greatest specificity, it had the lowest sensitivity compared to other Amsel’s criteria. The lowest specificity (46.6%) was observed in vaginal pH. Overall, the presence of over 20% clue cells in vaginal wet mount, with the sensitivity of 97.6% and specificity of 77.3%, was the best single Amsel’s criterion for *BV* diagnosis.

**Tablet 4:** Diagnostic accuracy of individual clinical criteria for the diagnosis of *bacterial vaginosis* among women attending research in Resalat Health Center of Amol (July 2013 - January 2014)

Amsel’s Criteria	SN (95% CI)	SP(%)(95% CI)	PPV (%)	NPV (%)	ACCURACY (%)
Vaginal pH	83.3 (69.5, 90)	46.6 (42.5, 61.5)	45.9	86.4	53.4
Amine test	54.0 (51.5, 78.5)	85.7 (78.4, 95.8)	68.4	79.6	74.4
Clue cells	97.6 (78.2, 95.6)	77.3 (95, 99.2)	75.2	78.2	84.9
Gray-White Discharge	86.7 (75.2, 95.1)	56.5 (47.5, 63.5)	44.9	90.1	68.1

N - Number; SN - Sensitivity; SP - Specificity; PPV - Positive predictive value; NPV - Negative predictive value.

## 4. Discussion

In the current study, the sensitivity, specificity, positive predictive value, negative predictive value, and accuracy of Amsel’s criteria were calculated as 91%, 91%, 86%, 94%, and 91%, respectively. Moreover, the presence of clue cells in vaginal wet mount and whiff tests had the greatest sensitivity (97.6%) and specificity (85.7%), respectively. In 2010, Ling et al. observed grayish white homogeneous vaginal discharge in 87% of women with *BV* (Ling et al., 2010). Simbar et al. treated women with either metronidazole or thyme and reported the prevalence of grayish white homogeneous vaginal discharge as 100% in both groups (Simbar, Azarbad, Mojab, & Alavimajd, 2008). According to our findings, the mentioned discharge had a sensitivity of 86.7% and a specificity of 56.5% in the diagnosis of *BV*. On the other hand, the presence of clue cells, an Amsel’s criterion with a sensitivity of 76.7% and a specificity of 92.4%, was the most valuable diagnostic criterion for *BV*. Clue cells are vaginal epithelial cells with granular, stippled appearance due to the attachment of bacteria (Amsel et al., 1983). Simoes et al. reported the high sensitivity (86%) and specificity (93%) of these cells in the diagnosis of *BV* (Simoes et al., 2006). Other studies have confirmed the presence of clue cells in vaginal discharge of 93% of patients with *BV*. The cells have also been found in vaginal wet mount of 93% of women with *BV* (Gutman, Peipert, Weitzen, & Blume, 2005). Likewise, Islam et al. affirmed the high sensitivity and specificity of clue cells in *BV* diagnosis (Islam, Safdar, & Malik, 2009). In the current study, the high sensitivity (97.6%) and specificity (77.3%) of these cells were calculated in the diagnosis of *BV*.

The whiff test (the release of a fishy odor upon adding one drop of 10% potassium hydroxide) is another criterion for *BV* diagnosis with a sensitivity of 33.9% and a specificity of 86.9% (Hallen, Pahlson, & Forsum, 1987). Hallen et al. evaluated clinical criteria on individuals presenting at clinics for sexually transmitted diseases. They found positive whiff test results in 95% of the patients (Hallen, Pahlson, & Forsum, 1987). Similarly, we determined the sensitivity and specificity of the test as 54.0% and 85.7%,

Vaginal pH≥4.5 is also an Amsel’s criterion with very high sensitivity (97%), but low specificity (26%) (Simoes et al., 2006). While vaginal pH normally falls between 3.8 and 4.2, it can change based on the activity of vaginal microflora (Ma, Forney, & Ravel, 2012). In addition to *BV*, trichomoniasis, cervical secretions, contact with semen, and application of lubricant gels can increase vaginal pH. Therefore, combining pH tests with other symptoms can enhance the accuracy of the test in diagnosis of various infectious conditions (Gutman, Peipert, Weitzen, & Blume, 2005). We found vaginal pH to have the lowest specificity (46.6%). Various factors such as simultaneous infections of vagina and cervical mucus might have influenced this criterion.

Rangari et al. in their study of 2013 reported Nugent scoring system had a higher sensitivity in diagnosing *BV* while Amsel’s criteria had less sensitivity and higher specificity. They concluded Amsel’s criteria without utilizing staining methods could be misleading (Rangari Amit, Parmjit, & Sharma, 2013). According to their study, by Amsel’s criteria false positive were 26.4% (Because of the high specificity) while 1.2% cases of *BV* were missed. Thus Nugent score can help in avoiding overestimating and further treatment of *BV*. This contrasts with the findings of our study. The present study showed Amsel criteria to have high specificity (91%) and sensitivity (91%) in *BV* diagnosis. Our findings showedAmsel criteria could be as good as Nugent scoring system at diagnosing this infection.

The specificity and sensitivity of Amsel’s criteria were respectively 96.5% and 78.0% in comparison with Nugent scoring system (Bhat, Kotigadde, & Shenoy, 2011). Taj et al. calculated the prevalence of *BV* as 62% by Amsel’s criteria and 78% by Gram staining. They reported the sensitivity, specificity, positive predictive value, and negative predictive value of Amsel’s criteria as 77%, 91%, 97%, and 53%, respectively (Taj, Nasir, Kahkashan, & Anjum, 2012). Although these findings are consistent with ours, Moussavi and Behrouzi suggested Amsel’s criteria to have low diagnostic validity with a sensitivity of 78%, a specificity of 88%, a positive predictive value of 95%, and a negative predictive value of 85% (Moussavi & Behrouzi, 2004). Since Gram staining is a reproducible and reliable method for *BV* diagnosis, the results obtained based on Amsel’s criteria have to be confirmed by Gram staining.

Dadhwal et al. reported the sensitivity, specificity, and positive and negative predictive values of Amsel criteria as 51.2%, 98.0%, 71.0%, and 95.5%, respectively (Dadhwal, Hariprasad, Mittal, & Kapil, 2010). Mengistie et al. compared various diagnostic methods for *BV* and suggested Amsel clinical criteria to have a sensitivity of 85.7% and a specificity of 98.0% compared with Nugent scoring system. They indicated the presence of clue cells as the individual Amsel’s criterion with the highest specificity and sensitivity. Moreover, the whiff test and pH were detected to have the lowest specificity and sensitivity, respectively. The researchers thus concluded that in the absence of Gram staining, Amsel’s criteria could be used as a practical method for diagnosis of *BV* (Mengistie, Woldeamanuel, Asrat, & Yigeremu, 2013).

## 5. Conclusion

The present study showed Amsel criteria to have high specificity and sensitivity in *BV* diagnosis. As this technique is highly efficient and requires low costs and time (due to the need for less equipment), If lab equipment is not available for diagnosing BV, Amsel’s criteria can be as good as Nugent scoring system at diagnosing this infection.
